# On the Nervous System, in Reply to Londinensis

**Published:** 1815-06

**Authors:** C. W. Smerdon

**Affiliations:** Clifton, Bristol


*For the Medical and Physical Journal.*

### *On the Nervous System, in Reply to Londinensis;* by Mr. SMERDON.

IN answer to queries which have been put to me, by your correspondent Londinensis, I beg leave to subjoin the following observations.

At the commencement of my remarks on the functions of the nervous system, I attempted to define the cause of muscular motion to be consequent on an action between nervous energy and the irritable principle; I considered that all parts, which contract on the application of stimuli, possess this principle, and that it is independent of actions and of organization: but I did not say living organization, nor can I conceive how such an inference could be drawn, since in another part I have admitted, that irritability may be either life or its essence.

Londinensis

Londinensis will admit, I suppose, that muscular fibre does not become disorganized immediately on losing its vitality; but, when a muscle is deprived of life, it also losing the property of contracting on the application of stimuli;* the arteries of a dead animal, when injected, with an irritating liquid, remain passive;† and the well known effect of galvanism on a sheep recently killed, which a few hours after the apparent death of the animal cannot be produced again, prove pretty evidently, that living muscles possess an irritable property, which cause them to contract on the application of a stimulating power, but which they retain only so long as they retain the living principle.

The knowledge of this contractile property in muscular fibre, is of great consequence to the surgeon: in his treatment of dislocations, it will point out to him the primary necessity of relaxing such muscles as are kept permanently contracted, by being placed in an unnatural position; and he will thus sometimes be enabled to overcome by art, what the most powerful mechanical force would only have rendered still more formidable. Hence too, amputation is indebted for much improvement, at least so far as the after-treatment is concerned; nor is it possible to make a good stump, especially if the thigh be the part operated on, if the surgeon neglect to provide against this peculiar law of nature.

The irritable principle is confined to parts which contract on the application of stimuli. The lungs, the liver, the kidnies, &c. have not this property, although they are plentifully supplied with nerves; hence in animals it is exclusively confined to the muscular fibre. It is probable, also, that this principle is the connecting medium between animals and plants, and that it is one cause of contractibility in those species of the latter which are called sensitive.

That matter cannot act where it is not, is an axiom which all men will acknowledge, who are not sceptically determined to deny the material existence of whatsoever is not cognizable to their senses. If, for instance, a deaf man were to affirm, that a combination between air and a few

---

* If this were owing to the deprivation of life, then irritability could not be distinct from the living principle; but all parts of an animal live, and are endowed with nerves, yet have not this property of contracting: in either case it is clear, that irritability is independent of organization.

† It would appear, by the experiments of Sir E. Home, that the alternate contractions of arteries are influenced, if not wholly occasioned, by the action of nervous energy on the irritability of their muscular coat.

tremulous strings could not possibly produce sound; or, if he were to deny the existence of sound altogether, unless he had ocular demonstrations of it, would any one who possesses the sense of hearing become a convert to his opinion? I consider the person who denies the existence of matter, because it can neither be seen nor felt, to be the same sort of reasoner as this deaf man. For my own part, I conceive that causes must be always adequate to their effects; and when I see the most durable work of art or nature rent in twain by an invisible agent, or the spontaneous motion or contraction of the muscles of animals which we know is lost when their connection with the brain is cut off; I feel persuaded that such phenomena cannot be brought about but by something that is material.

Instances are not wanting to prove, that the brain, considered as an organ, is not a sensitive part; considerable portions of it have been lost by sloughing, during which the patient complained of but little pain, was sensible and ultimately recovered. A case similar to this was lately published in one of the periodical journals. It is only when the brain is inflamed, when its functions are disturbed, or the matter which these functions produce irritated, that it becomes exquisitely sensible; and then the effects which are ascribed to it are many times increased. The ideas of a patient labouring under phrenitis succeed each other with astonishing rapidity. The various passions of the mind, by turns, engross for a few moments the whole of his attention, and these are strongly pourtrayed by the continual changes of the countenance. The energies of his mind are equalled by the powers of the body, for the increase of strength which his muscles receive is truly astonishing; so that a weakly man may, during a paroxysm of phrenitis, require the efforts of several to confine him in bed. If we add to these symptoms of the first stage of an inflamed brain, the debility of the organ, (as evinced by torpor, lassitude, and idiotism,) which supervenes on the accession of the second stage, we shall, I think, have sufficient evidence for believing, that the brain is governed by the same laws as the other organs of the body, which secrete fluids each peculiar to itself.

The increase of muscular power consequent on an excited brain, and the extreme weakness of the muscles which follows this excitement when the organ is in a contrary state, are evidences in favour of a nervous fluid; by this term I mean a something generated in the brain of a highly stimulating nature, which, being sent to the muscles through the medium of the nerves, produces certain effects, which are increased when the brain is excited, diminished when this organ is in a state of debility; but what this something is, by what means produced, whether by a secretory process analogous to the other organs, or by an agency which at present we know nothing of, I shall not presume to give an opinion.

Pressure, in proportion to its quantity, retards the functions of the brain, at least so far as voluntary motion is concerned; but pressure, however long continued, does not necessarily destroy the vitality of the organ, nor hinder the common actions of the body from going on in it which are required for its due nourishment;* hence we learn, that, in common with the secreting organs, two actions go on in the brain, the one necessary for its vitality, and the other for the development of its functions. These facts are still further proofs of the existence of a nervous fluid; they shew us that the brain when pressed upon, continues to live and to be nourished, while its connection with the muscles, through the medium of nerves, is precisely the same as before the pressure; notwithstanding which, the muscles lose their contractile power and remain passive; remove this pressure, and what follows? the brain, if not disorganized, resumes its functional actions, and the muscles again become obedient to the will.

The nervous fluid, then, is the connecting medium between the willing power of the mind and the moving fibres of the body; by the first the design is conceived which the others execute; cut off this connection, either by obstructing the passage of the fluid, or by destroying the functions of the brain, and the muscles will become inactive and apparently lose their contractile property.

We need not go far for evidence to prove, that a most powerful agent can exist in a state not cognizable to our senses; I mean the matter which emanates from an excited electrical machine or a galvanic battery; for matter (I again repeat) cannot act where it is not, and consequently when I see a body violently acted upon and decomposed by simple contact with a wire, the other end of which is connected with a peculiar arrangement of two metals and water, I cannot help being satisfied that this phenomenon is brought about by a material agent, and which passes from that arrangement, by means of the wire, to the body acted upon.

---

* In proof of this, I beg Londinensis will recall to his recollection, the case of the sailor who was trephined in Guy's Hospital, and who ultimately recovered, after having been in a state of insensibility for more than twelve months.

In answer to the last query, I shall say but little. I fully agree with the distinction which Mr. Abernethy has drawn between hypothesis and theory; and, strictly speaking, I have not the vanity to suppose, that any thing which I have written amounts to more than an hypothesis.

*Clifton, Bristol;* C. W. SMERDON.

*April* 8, 1815.

